# Metabolic Consequences of Neuronal HIF1α-Deficiency in Mediobasal Hypothalamus in Mice

**DOI:** 10.3389/fendo.2021.668193

**Published:** 2021-10-18

**Authors:** Azmat Rozjan, Weibi Shan, Qiaoling Yao

**Affiliations:** ^1^ Department of Physiology, School of Basic Medical Sciences, Xinjiang Medical University, Urumqi, China; ^2^ Department of Dermatology, The First Affiliated Hospital of Xinjiang Medical University, Urumqi, China

**Keywords:** HIF1α in the hypothalamus, obesity, glucose, lipid metabolism, insulin resistance

## Abstract

**Objective:**

This study aims to investigate whether hypoxia-inducible factor 1α (HIF1α) in the neurons of the mediobasal hypothalamus is involved in the regulation of body weight, glucose, and lipid metabolism in mice and to explore the underlying molecular mechanisms.

**Methods:**

HIF1α*^flox/flox^* mice were used. The adeno-associated virus that contained either cre, GFP and syn, or GFP and syn (controls) was injected into the mediobasal hypothalamus to selectively knock out HIF1α in the neurons of the mediobasal hypothalamus. The body weight and food intake were weighed daily. The levels of blood glucose, insulin, total cholesterol (TC), triglyceride (TG), free fatty acid (FFA), high-density lipoprotein (HDL), and low-density lipoprotein (LDL)were tested. Intraperitoneal glucose tolerance test (IPGTT) was performed. The insulin-stimulated Akt phosphorylation in the liver, epididymal fat, and skeletal muscle were examined. Also, the mRNA expression levels of HIF1α, proopiomelanocortin (POMC), neuropeptide Y (NPY), and glucose transporter protein 4 (Glut4) in the hypothalamus were checked.

**Results:**

After selectively knocking out HIF1α in the neurons of the mediobasal hypothalamus (HIF1αKOMBH), the body weights and food intake of mice increased significantly compared with the control mice (*p* < 0.001 at 4 weeks). Compared with that of the control group, the insulin level of HIF1αKOMBH mice was 3.5 times higher (*p* < 0.01). The results of the IPGTT showed that the blood glucose level of the HIF1αKOMBH group at 20–120 min was significantly higher than that of the control group (*p* < 0.05). The serum TC, FFA, HDL, and LDL content of the HIF1αKOMBH group was significantly higher than those of the control group (*p* < 0.05). Western blot results showed that compared with those in the control group, insulin-induced AKT phosphorylation levels in liver, epididymal fat, and skeletal muscle in the HIF1αKOMBH group were not as significantly elevated as in the control group. Reverse transcription-polymerase chain reaction (RT-PCR) results in the whole hypothalamus showed a significant decrease in Glut4 mRNA expression. And the mRNA expression levels of HIF1α, POMC, and NPY of the HIF1αKOMBH group decreased significantly in ventral hypothalamus.

**Conclusions:**

The hypothalamic neuronal HIF1α plays an important role in the regulation of body weight balance in mice under normoxic condition. In the absence of hypothalamic neuronal HIF1α, the mice gained weight with increased appetite, accompanied with abnormal glucose and lipid metabolism. POMC and Glut4 may be responsible for this effect of HIF1α.

## Background

Obesity refers to a metabolic disease in which the energy intake exceeds the energy expenditure, leading to increased body weight and body fat content. In 2015, 107.7 million children and 603.7 million adults were obese in the world, and about 1.9 billion were overweight (1, 2). The epidemics of obesity and obesity-related diseases, such as dyslipidemia, hypertension, and type 2 diabetes, are rapidly increasing worldwide (3, 4). Researchers never stopped exploring the treatment of obesity, but to date, no absolute safe and effective weight loss method or drug have been found. Therefore, studying the pathogenesis of obesity from different perspectives and finding possible treatments are still necessary.

The hypoxia-inducible factor (HIF) is a transcription factor that is stably expressed in hypoxic cells and participates in various adaptive responses (5, 6). Three isoforms of HIF, namely, HIF1α, HIF2α, and HIF3α, are identified. Studies showed that HIF1α and HIF2α are stably expressed in the brain, including hypothalamus under normoxic condition (7, 8). Moreover, studies showed that the hypothalamic HIFs play an important role in appetite regulation in mice. Studies demonstrated that increased hypothalamic glucose level activates HIF and inhibits feeding in mice (7). The knockdown of both HIF1α and HIF2α in proopiomelanocortin (POMC) neurons or arcuate nucleus (ARC) results in pronounced weight gain under high-fat diet (HFD) (7, 9). The HIF2α expression decreases with the aging of the mice, and the deletion of HIF2α in hypothalamic POMC neurons can lead to age-dependent weight gain and increased body fat content in mice with mild glucose intolerance and insulin resistance (8). These studies suggested that HIFs, especially HIF2α, in hypothalamic ARC, precisely POMC neurons may be involved in appetite regulation and body weight regulation in mice fed with HFD. However, it is not clear whether hypothalamic neuronal HIF1α itself plays a role in body weight regulation in mice, especially under normal diet and whether it affects the glucose and lipid metabolism.

Therefore, in our study, HIF1α*^flox/flox^* mice were selected, and adeno-associated viruses containing *cre* fragments and neuron-specific promoter *syn* were injected into mediobasal hypothalamus to knock out HIF1α selectively in the neurons of the mediobasal hypothalamus. The body weight and food intake of the mice are recorded under chow diet. Also, the glucose metabolism and the lipid metabolism of the mice were examined. As a first step to understand the role of HIF1α in body weight control, we aim to identify the metabolic phenotype of neuronal HIF1α-deficiency in mediobasal hypothalamus in mice.

## Materials and Methods

### Animals

Breeding pairs of HIF1α*^flox/flox^* mice were gifts from Polotsky Laboratory (10) of Johns Hopkins University and bred at the Animal Experimentation Center of Xinjiang Medical University. Male HIF1α*^flox/flox^* mice aged 6–8 weeks and weighing approximately 23 g were used for the study. Male C57BL/6J mice aged 6–8 weeks were used to test the effect of viruses on body weight. All mice had free access to water and were housed in a standard specific pathogen free (SPF)-grade laboratory environment at 22°C–23°C with a 12-h light/dark cycle (09:00–21:00/21:00–09:00). Mice were anesthetized by intraperitoneal injection of 4% chloral hydrate (0.13 ml/10 g) for all surgeries. After virus injection, up to five mice were kept in one cage on chow diet (9.4% kcal from fat) (11), and cages were changed twice a week. The daily food intake of mice is calculated by cage: the total food intake of the cage/the number of mice. All animal experiments were approved by the Animal Ethics Committee of Xinjiang Medical University and conducted in accordance with the guidelines established by this committee.

### Mediobasal Hypothalamic Injection of Virus to Knock Down HIF1α

For both HIF1α*^flox/flox^* mice and C57BL/6J mice, AAV-hSyn-cre-GFP (cre) and AAV-hSyn-GFP (GFP) (serotype 9, GENE, AAV9CON323) were injected into the mediobasal hypothalamus by stereotaxic injection at the following coordinates: 1.5 mm form bregma; midline, ± 0.5 (both sides); and dorsoventral, −5.8 (from cranial surface). Each side was given an injection of 0.5 µl virus at a concentration of 1.45 E^+13^ v g/ml.

### Experimental Design

The mice with significant weight gain were divided into three batches 28–35 days after the virus injection. In the first batch, the brain of the mice was harvested after whole body perfusion for frozen section. Intraperitoneal glucose tolerance test (IPGTT) and insulin signaling pathway were examined in the mice of the second batch. The blood and the fresh hypothalamus were harvested in the mice of the third batch for future use.

### Frozen Sections to Observe the Location of Injections and Expression of Viruses

The HIF1α*^flox/flox^* mice were anesthetized and rapidly perfused with sterile normal saline and 4% paraformaldehyde 28–35 days after virus injection. The brains were carefully removed, postfixed in 4% paraformaldehyde at 4°C for overnight. The next morning, the brains were cytoprotected in 20% sucrose solution at 4°C for 24 h. Then, the brains were frozen and stored at −80°C. Whole brains were fixed with the O.C.T. compound and 30 µm of sections were cut and stored in PBS buffer. Then the brain slices were mounted on the slide. After sealing with antifade solution (Solarbio, S2100, Beijing, China), the injection location and virus expression were observed and pictured by confocal microscope (Leica, SP8, Wetzlar, Germany) with full-slice scanning using ×10 magnification.

### IPGTT

IPGTT were performed after 6 h fasting. The glucose solution (1 g/kg) (BIOFROXX, 1179GR500, Einhausen, Germany) was intraperitoneally injected into the mice after testing blood glucose at baseline. The blood glucose was measured at the tip of the tail using a handheld glycemic meter (Roche Accu-Chek, Active, Indianapolis, IN, USA) at 10, 20, 30, 60, 90, and 120 min after injection of glucose.

### Biochemical Measurements

The triglyceride (TG) assay kit (Elabscience, E-BC-K238, Wuhan, China) was used to detect TG content in serum. The total cholesterol (TC) ELISA kit (Elabscience, E-BC-K179) was used to detect the content of TC in serum. The mouse free fatty acids (FFA) ELISA kit (TW-reagent) was used to detect the content of FFA in serum. The glucose content in serum was detected using the mouse glucose ELISA Kit (Elabscience, E-BC-K268). The serum insulin content was measured using the mouse insulin ELISA Kit (Raybio, ELM-Insulin, Peachtree Corners, GA, USA).

### Insulin Signaling Pathway Detection

Insulin signaling pathway activation in mice was performed by administering 5 U/kg insulin intraperitoneally 15 min prior to the sacrifice of the mice. In control mice, saline was injected. Liver, epididymal fat, and skeletal muscle tissue were collected, snap frozen in liquid nitrogen, and stored at −80°C. Tissues were homogenized through the Ripa buffer (ThermoFisher, VH310061, Waltham, MA, USA). The gels for Western blot were made by a gel-making kit (Solarbio, PC0020). Proteins (30 μg) were applied to each lane. For total and phosphorylated Akt measurements, we used antibody phospho-Akt (Ser473) (D9E) XP rabbit mAb (Cell Signaling, #4060, Danvers, MA, USA) and Akt (pan) (11E7) rabbit mAb (Cell Signaling, #4685). The optical densities of bands were measured using the ImageJ software. Insulin signaling was assessed by calculation of the ratio of pAkt to actin or total Akt after insulin injection.

### Quantitative RT-PCR of HIF1α, POMC, Neuropeptide Y, and Glut4

The total RNA was extracted from the homogenized hypothalamus by using TRIzol (Takara, 15596026, Ambion, Austin, TX, USA), and the RNA was reverse transcribed into cDNA by using rapid reverse transcription kits (RR047A, Kusatsu, Japan). Real-time fluorescence quantitative PCR was performed (Takara, RR820A). The amplification reaction conditions were as follows: stage 1: one cycle at 95°C for 30 s; stage 2: 40 cycles at 95°C for 5 s and 60°C for 34 s; stage 3: one cycle at 95°C for 15 s, 60°C for 1 min, and 95°C for 15 s. The CT values of the samples were measured using the Applied Biosystems 7500 Real-time PCR System. The relative expression level was calculated using 18S as reference, and the 2^−ΔΔCt^ method was used to analyze the data. The primers of the target mRNAs are as follows: HIF1α: sense, 5′-CAGCAAGATCTCGGCGAAGC-3′; antisense, 5′-TGATGGTGAGCCTCATAACAGA-3′. POMC: sense, 5′-CAAGGACAAGCGTTACGGTG-3′; antisense, 5′-GGGGCCTTGGAATGAGAAG-3′. Neuropeptide Y (NPY): sense, 5′-AGAAAACGCCCCCAGAACAA-3′; antisense, 5′-TAGTGGTGGCATGCATTGGT-3′. Glucose transporter protein 4 (Glut4): sense, 5′-CCAACAGCTCTCAGGCATCA-3′; antisense, 5′-CCGAGACCAACGTGAAGA-3′. 18S: sense, 5′-TTGACGGAAGGGCACCACCAG-3′; antisense, 5′-GCACCACCACCCACGGAATCG-3′.

### Statistical Analyses

Mean ± SEM was used to calculate the results, and two-sided unpaired *t*-tests were used for both data sets. Pearson correlation tests for correlation between variables and normal distributions were performed using IBM SPSS Statistics 26 analysis software, as well as repeated measures ANOVA for weight and food intake for HIF1αKOMBH, C57BL/6J, and controls, and Bonferroni was used for multiple comparisons. GraphPad Prism 9 was used to calculate the area under the curve. Data are expressed as mean ± SEM, and *p* < 0.05 was considered statistically significant.

## Results

### Localization and Expression of Viruses

**Figure 1:**
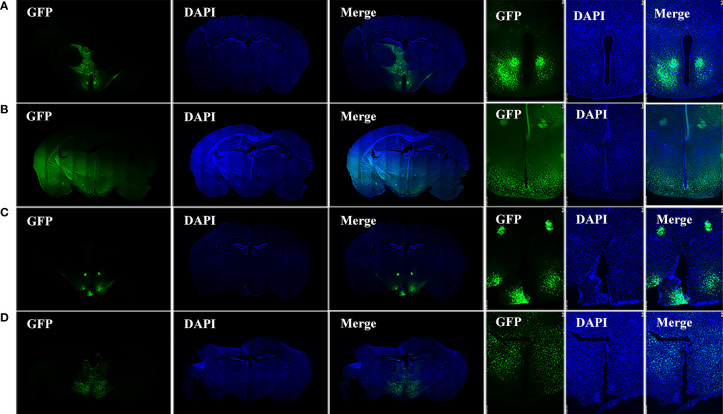
Injection site of viruses. **(A)** Brain sections of HIF1α*^flox/flox^* mice injected with AAV-hSyn-GFP virus in the mediobasal hypothalamus. **(B)** Brain sections of HIF1α*^flox/flox^* mice injected with AAV-hSyn-cre-GFP virus in the mediobasal hypothalamus. **(C)** Brain sections of wild-type C57BL/6J mice injected with AAV-hSyn-GFP virus at the mediobasal hypothalamus. **(D)** Brain sections of wild-type C57BL/6J mice injected with AAV-hSyn-cre-GFP virus at the medial mediobasal hypothalamus.

Frozen sections of the brains were collected 28–35 days after HIF1α*^flox/flox^* mice were injected with AAV-hsyn-GFP and AAV-hsyn-cre-GFP to observe the locations and expressions of virus injection. The green fluorescence in **Figures 1A, B** are the expression locations of AAV-hsyn-GFP and AAV-hsyn-cre-GFP, respectively. The green fluorescence in **Figures 1C, D** are the expression locations of AAV-hsyn-GFP and AAV-hsyn-cre-GFP in wild-type (WT) C57BL/6J mice. The injection and expression sites of viruses can be seen around the third ventricle, in the mediobasal hypothalamus. Due to variations of the injections, the small differences of expression positions can be seen in the figure. The leakage of the viruses can also be seen through the injection route.

### Increased Body Weight and Food Intake in HIF1αKOMBH Mice

**Figure 2:**
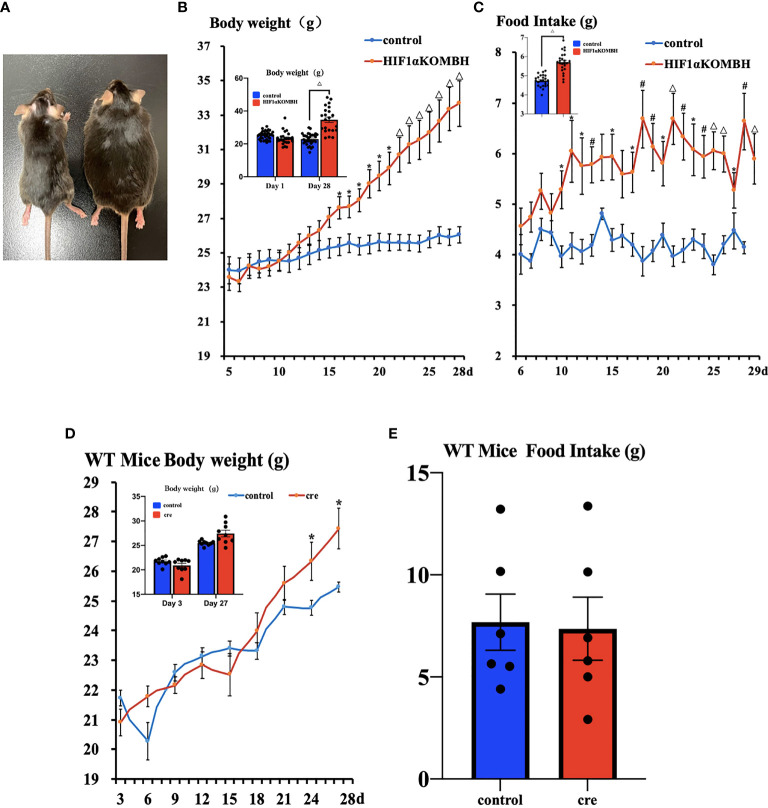
Changes in the body weight and food intake of HIF1α*^flox/flox^* mice injected with AAV-hSyn-cre-GFP and AAV-hSyn-GFP at the mediobasal hypothalamus. **(A)** HIF1α*^flox/flox^* mice injected with AAV-hSyn-GFP (left) and AAV-hSyn-cre-GFP (right) at the mediobasal hypothalamus for 28 days. **(B)** Changes in body weight of HIF1α*^flox/flox^* mice after injections of AAV-hSyn-cre-GFP and AAV-hSyn-GFP at the mediobasal hypothalamus. **(C)** Effects of AAV-hSyn-cre-GFP and AAV-hSyn-GFP injections at the medial mediobasal hypothalamus of HIF1α*^flox/flox^* mice on food intake. **(D)** Changes in body weight of wild-type C57BL/6J mice after injections of AAV-hSyn-cre-GFP and AAV-hSyn-GFP at the mediobasal hypothalamus. **(E)** Effects of AAV-hSyn-cre-GFP and AAV-hSyn-GFP injections at the mediobasal hypothalamus of wild-type C57BL/6J mice on food intake. ^*^ *p* < 0.05, ^#^ *p* < 0.01, and ^△^ *p* < 0.001.

No significant difference in mean body weight was found between the HIF1αKOMBH and control groups before virus injection. On 16 to 28 days after virus injection, the body weights of HIF1αKOMBH mice increased significantly compared with the control mice (**Figure 2B**). The repeated measures ANOVA analysis showed that the interaction term was statistically significant, *F* (23, 897) = 17.880, *p* < 0.05, bias *η* ^2^ = 0.314, suggesting an interaction effect of group and time change on body weight in mice. By comparing the difference in body weight of mice seen in the two groups, the results showed that *F* = 7.006, *p* < 0.05, deviation *η* ^2^ = 0.152, indicating that the difference in body weight of mice in the two groups was significant. Twenty-eight days after virus injection, the mean body weight of mice in the HIF1αKOMBH group reached 33.68 ± 1.43 g (*n* = 26), which was 1.3 times that of the control mice (26.01 ± 0.48 g, *n* = 15; **Figure 2B**). The maximum body weight of mice in the HIF1αKOMBH group reached 46.2 g (**Figure 2A**). For wild-type C57BL/6J mice, significant increases in body weight on days 24 and 27 after virus injection were seen in the cre group compared with the control group (*p* < 0.05) (*n =* 9, **Figure 2D**). However, the maximum body weight of the C57BL/6J mice in the cre group on day 27 after virus injection was only 30.9 g. The two-way ANOVA analysis showed that the interaction term for wild-type C57BL/6J mice in this study was statistically significant, *F* (8, 128) = 79.076, *p* < 0.05, deviation *η* ^2^ = 0.832, but there was no significant difference in weight between the two groups (*F* = 0.825, *p* > 0.05, deviation *η* ^2^ = 0.049).

The food intake of HIF1α*^flox/flox^* mice injected with AAV-hsyn-cre-GFP significantly increased compared with that of the control mice. The two-way ANOVA analysis showed that the interaction term was statistically significant, *F* (23, 575) = 2.389, *p* < 0.05, bias *η* ^2^ = 0.087, suggesting an interaction effect of group and time change on the change of food intake. Statistical significance was observed between the two groups of mice from days 10 to 13, 15, and 17–29 (*p* < 0.05, *p* < 0.01, and *p* < 0.001, respectively; **Figure 2C**). The results of the comparison of the difference in the amount of food eaten by the mice between the different groups showed a significant difference in the weight of food eaten by the mice between the two groups (*F* = 10.914, *p* < 0.05, deviation *η* ^2^ = 0.304). For WT C57BL/6J mice, the number of days and group interaction had a significant effect on mouse feeding (*F* (5, 75) = 7.476, *p* < 0.05, deviation *η* ^2^ = 0.333). However, there was no difference in mouse feeding between groups (*F* = 0.044, *p* > 0.05, deviation *η* ^2^ = 0.003) (*n* = 9, **Figure 2E**).

### Blood Glucose and Insulin Levels in HIF1αKOMBH Mice

**Figure 3:**
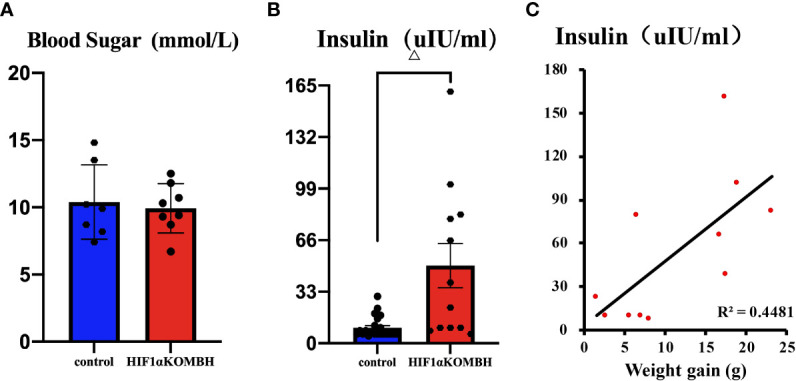
Changes in blood glucose and insulin content and the correlation between serum insulin content and body weight of mice after injections of AAV-hSyn-GFP and AAV-hSyn-cre-GFP in the mediobasal hypothalamus of HIF1α*^flox/flox^* mice. **(A)** Serum blood glucose levels of HIF1α*^flox/flox^* mice injected with AAV-hSyn-cre-GFP and AAV-hSyn-GFP. **(B)** Serum insulin contents of HIF1α*^flox/flox^* mice injected with AAV-hSyn-cre-GFP and AAV-hSyn-GFP. **(C)** Correlation analysis between serum insulin content of HIF1α*^flox/flox^* mice and body weight changes after injections of AAV-hSyn-GFP and AAV-hSyn-cre-GFP (^△^ *p* < 0.001).

Thirty days after AAV-hsyn-GFP and AAV-hsyn-cre-GFP injection in the mediobasal hypothalamus of HIF1α*^flox/flox^* mice, serum insulin and fasting blood glucose were detected. No significant differences were shown in fasting blood glucose level between HIF1αKOMBH mice (*n* = 8) and control mice (*n* = 7) (**Figure 3A**). However, the serum insulin level of HIF1αKOMBH mice reached 49.70 ± 14.18 UIU/ml (*n* = 12), which was 4.9 times higher than that of the control mice (*n* = 23, *p* < 0.01, **Figure 3B**). The correlation analysis in HIF1αKOMBH mice showed that the serum insulin level was positively correlated with weight gain (*p* < 0.05, *r* = 0.669, *n* = 11; **Figure 3C**).

### Impaired Glucose Tolerance in HIF1αKOMBH Mice

**Figure 4:**
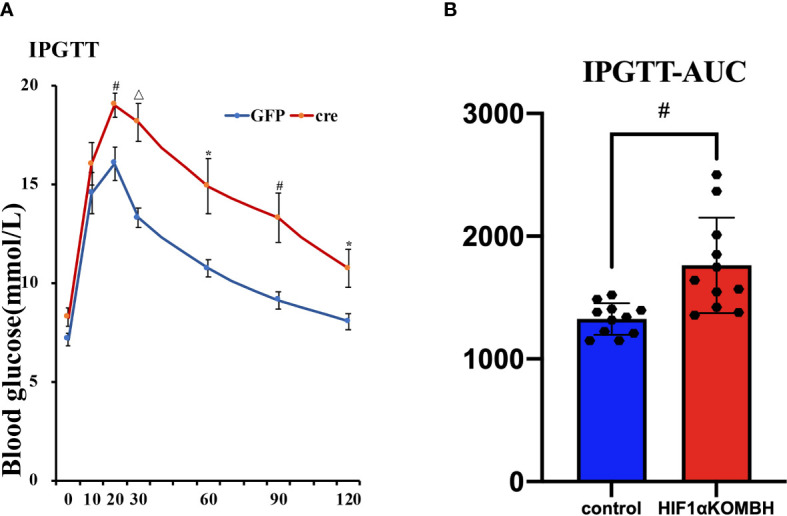
Effects of AAV-hSyn-GFP and AAV-hSyn-cre-GFP injections into the mediobasal hypothalamus HIF1α*^flox/flox^* mice on the intraperitoneal glucose tolerance (IPGTT) and subsurface area (AUC). Changes in **(A)** glucose tolerance, **(B)** area under the glucose tolerance test curve. (^*^ *p* < 0.05, ^#^ *p* < 0.01, and ^△^ *p* < 0.001).

IPGTT was performed 28–35 days after virus injection to investigate the glucose tolerance of mice. The results showed that both groups had peak blood glucose levels 20 min after glucose injection. There were no significant differences for the baseline glucose level of the two groups, similar with the fasting glucose level we tested before. However, HIF1αKOMBH mice had higher blood glucose levels at each time point after glucose injection than control mice, and the statistical significances were seen at 20, 30, 60, 90, and 120 min after glucose injection (*n* = 11; *p* < 0.05, *p* < 0.01, and *p* < 0.001, **Figure 4A**). The area under the curve (AUC) of the HIF1αKOMBH knockout mice (1,880.38 ± 138.69) was 1.2 times higher than that of the control mice (1,350.38 ± 49.51, *p* < 0.01; **Figure 4B**).

### Attenuated Insulin Signaling Pathway in HIF1αKOMBH Mice

**Figure 5:**
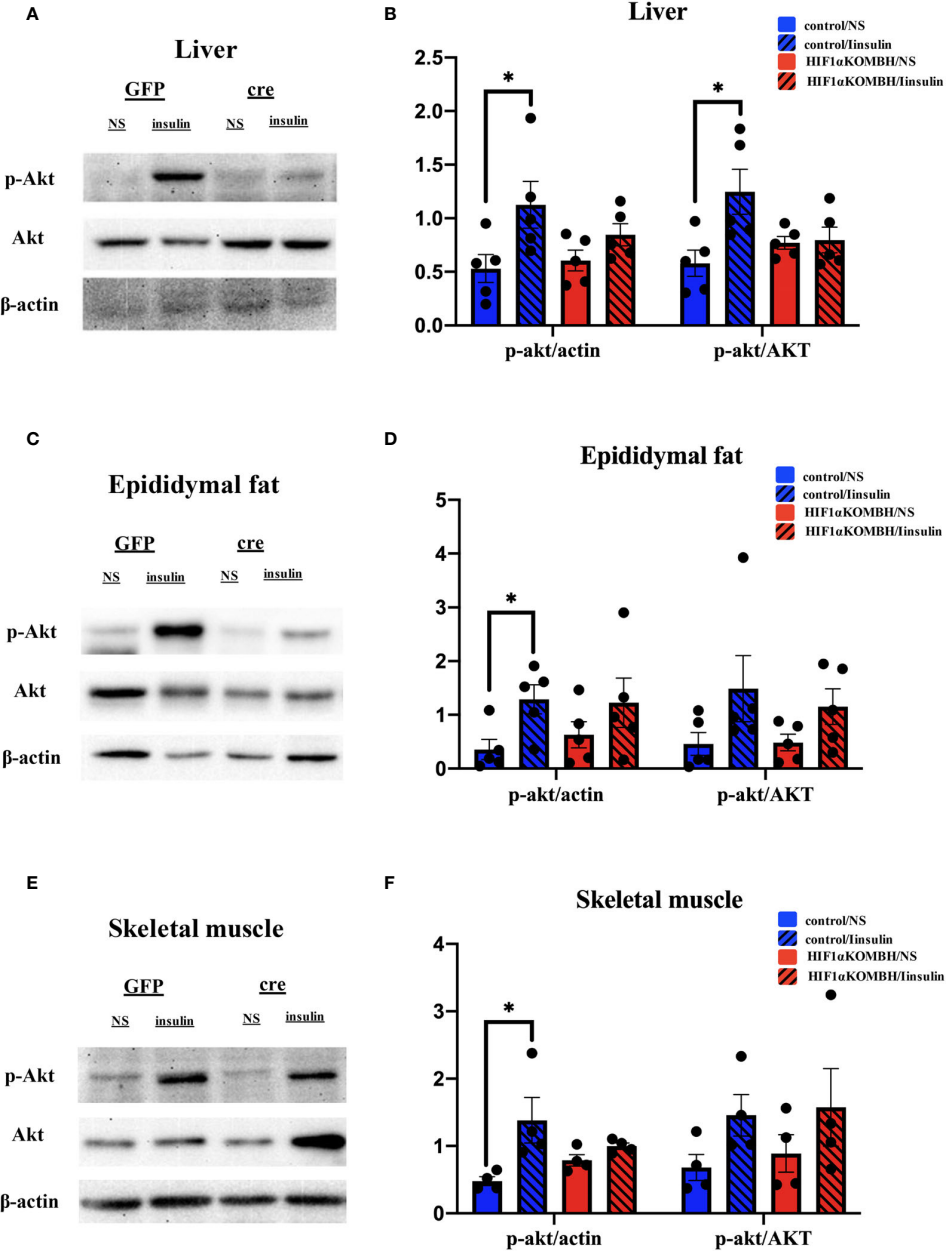
Effects of AAV-hSyn-GFP and AAV-hSyn-cre-GFP injections into the mediobasal hypothalamus of HIF1α*^flox/flox^* mice on insulin signaling pathway. **(A)** Detection of the p-Akt and Akt protein expression levels in the mouse liver after AAV-hSyn-GFP and AAV-hSyn-cre-GFP injection into the mediobasal hypothalamus of HIF1α*^flox/flox^* mice. **(B)** Quantitative analysis results of p-Akt, Akt, and actin protein expression in the liver of HIF1α*^flox/flox^* mice after injections of AAV-hSyn-GFP and AAV-hSyn-cre-GFP into the mediobasal hypothalamus. **(C)** Expression of p-Akt and Akt protein in mouse epididymal fat detected after injections of AAV-hSyn-GFP and AAV-hSyn-cre-GFP into the mediobasal hypothalamus of HIF1α*^flox/flox^* mice. **(D)** Quantitative analysis results of p-Akt, Akt, and actin protein expression in the epididymal fat of HIF1α*^flox/flox^* mice after injections of AAV-hSyn-GFP and AAV-hSyn-cre-GFP into the mediobasal hypothalamus. **(E)** Expression of p-Akt and Akt protein in mouse skeletal muscle detected after injections of AAV-hSyn-GFP and AAV-hSyn-cre-GFP into the mediobasal hypothalamus of HIF1αflox/flox mice. **(E)** Quantitative analysis results of p-Akt, Akt and actin protein expression in the skeletal muscle of HIF1α*^flox/flox^* mice after injections of AAV-hSyn-GFP and AAV-hSyn-cre-GFP into the mediobasal hypothalamus (^*^ *p* < 0.05).

In control mice, p-Akt protein expression significantly increased in the liver, epididymal fat, and skeletal muscle in insulin injection group (*n* = 5; **Figure 5**), which implied a normal insulin signaling pathway in these mice. However, there was no significant difference in Akt phosphorylation in liver, epididymal fat, and skeletal muscle of HIF1αKOMBH mice between insulin injection group and saline injection group (**Figure 5**), indicating the impaired insulin sensitivity in HIF1αKOMBH mice.

### Abnormal Lipid Metabolism in HIF1αKOMBH Mice

**Figure 6:**
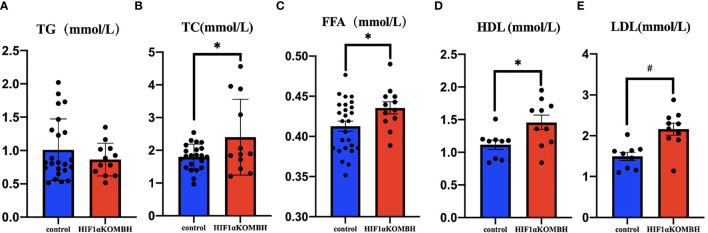
Changes in triglyceride, total cholesterol, free fatty acid, high-density lipoprotein and low-density lipoprotein content in the serum of HIF1αKOMBH mice. **(A)** Serum triglyceride, **(B)** serum total cholesterol, **(C)** free fatty acid contents, **(D)** high-density lipoprotein, and **(E)** low-density lipoprotein (^*^ *p* < 0.05, ^#^ *p* < 0.01).

The investigation of TC, TG, FFA, high-density lipoprotein (HDL), and low-density lipoprotein (LDL) levels in the serum of HIF1αKOMBH mice showed that lipid metabolism was also abnormal in HIF1αKOMBH mice. No significant difference was found in the serum TG content between the two groups (**Figure 6A**). The serum total cholesterol and FFA level in HIF1αKOMBH mice was significantly higher than control mice (*p* < 0.01 and *p* < 0.05, respectively, **Figures 6B, C**). For lipoprotein levels, the HDL and LDL content in HIF1αKOMBH mice was also significantly higher than those in control mice (*p* < 0.01 and *p* < 0.05, respectively, **Figures 6D, E**).

### Changes in the Expression of Protein and Appetite-Related Neuropeptides Associated With Systemic Energy Homeostasis in HIF1αKOMBH Mice

**Figure 7:**
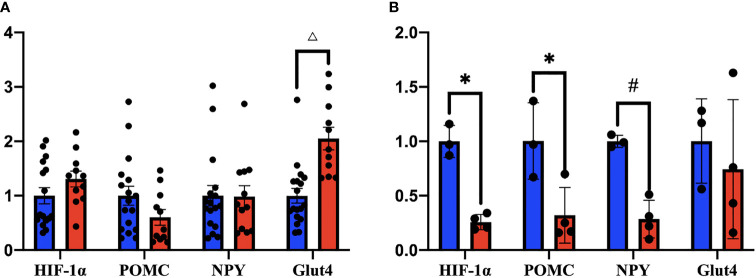
mRNA expression levels of HIF1α, POMC, NPY, and Glut4 in the hypothalamus of HIF1α*^flox^* mice after injection of AAV-hSyn-cre and AAV-hSyn-GFP (^*^ *p* < 0.05, ^#^ *p* < 0.01, and ^△^ *p* < 0.001). **(A)** Changes in the expression levels of HIF1α, POMC, NPY, and Glut4 mRNA in the whole hypothalamus. **(B)** Changes in HIF1α, POMC, NPY, and Glut4 mRNA expression levels in the ventral hypothalamus.

Fresh hypothalamus were harvested 28–35 days after virus injections to detect the mRNA expression of HIF1α, POMC, NPY, and Glut4 by RT-PCR. Tests on the whole hypothalamus showed no significant changes in HIF1α (*n* = 16), POMC (*n* = 17), and NPY (*n* = 17) between two groups. However, a 2.05 times increase in Glut4 mRNA levels compared with the controls were seen (*n* = 18) (**Figure 7A**). While tests on the ventral hypothalamus showed that the expression of HIF1α in HIF1αKOMBH mice (*n* = 4) was 74% less than the control group (*n* = 3) (*p* < 0.05), indicating that the neuronal HIF1α knockout in mediobasal hypothalamus of mice was successful. In ventral hypothalamus, the expression of POMC mRNA and NPY mRNA in HIF1αKOMBH mice (*n* = 4) was significantly reduced (*p* < 0.05) compared with the control group (*n* = 3), but the Glut4 expression in the hypothalamus of HIF1αKOMBH mice (*n* = 4) was not significantly different from the control group (*n* = 3) in ventral hypothalamus (**Figure 7B**).

## Discussion

In the central nervous system, the hypothalamus is the basic center for the regulation of energy metabolism and has a powerful regulatory role in energy intake, energy expenditure, and weight control (12–15), but the underlying mechanisms are unclear. Our study showed that HIF1α in neurons of mediobasal hypothalamus plays an important role in the regulation of body weight in mice under normoxic conditions. In the absence of HIF1α in neurons of mediobasal hypothalamus, the mice gained weight with increased food intake, accompanied with abnormal glucose and lipid metabolism.

The hypothalamus senses and regulates many signals of biometabolic dynamics (16, 17). In food-induced obesity, the whole-body energy homeostasis is disrupted (18), which may be related to abnormalities in hypothalamic neurons, e.g., NPY/agouti gene-related peptidergic neurons stimulating POMC/cocaine- and amphetamine-induced transcriptergic neurons (19–21). Conditional knockout of HIF in mouse hypothalamic POMC neurons using genetic methods and the knockout of HIF1β in the hypothalamic ARC have not revealed significant changes in feeding and body weight in mice fed with chow diet, but a significant increase in feeding and body weight is observed in those mice fed with HFD (7). In the present study, adeno-associated viruses containing *syn* (neuron-specific promoter) and *cre* fragments were injected into mediobasal hypothalamus of mice by stereotaxic injection, and HIF1α was conditionally knocked out in this site, confirmed by RT-PCR. Although we knocked out only HIF1α, rather than the HIF precursor, the body weight and food intake of mice with the chow diet increased significantly (*p* < 0.05), suggesting that neuronal HIF1α itself in mediobasal hypothalamus could be important for body weight and appetite control, although part of the reason may be due to broad regional HIF1α knockout neurons in the hypothalamus in our study. In addition to the hypothalamic POMC neurons and ARC region, HIF1α in other parts of the hypothalamic neurons may be involved in the regulation of body weight as well. In our results, the increase in body weight occurred earlier than the increase in food intake, suggesting that HIF1α in neurons of mediobasal hypothalamus might affect both the appetite and metabolic rate in mice. To exclude the effect of AAV-cre on body weight and food intake, we injected the same viruses in WT C57BL/6J mice. We did see the increase of body weight on 24 and 27 days after virus injection, but the increase of body weight was much less than HIF1α*^flox/flox^* mice. Moreover, the increase in food intake was not seen in WT mice. These results confirmed that the prominent weight gain and increased food intake in HIF1αKOMBH mice was due to loss of neuronal HIF1α in mediobasal hypothalamus.

Studies found that the loss of HIF1α in the vascular endothelial cells of mice leads to a significant increase in the fasting glucose level, slow insulin response after intravenous glucose injection, delayed glucose clearance in the blood, and significantly impeded glucose absorption by the brain and heart (22). These results indicated that HIF1α plays an important role in glucose metabolism. Other studies showed that HIF induction can enhance hypothalamic glucose sensing (7), and the inhibition of hypothalamic HIF1 lead to glucose intolerance and increased serum insulin level (8). In our study, impaired glucose metabolism was present in HIF1αKOMBH mice. Although there were no significant changes in blood glucose levels in HIF1αKOMBH mice, the serum insulin levels of HIF1αKOMBH mice were significantly increased, suggesting that mice with neuronal HIF1α knockout in the mediobasal hypothalamus developed insulin resistance. In addition, the normal blood glucose levels might be the result of increased insulin secretion. The results of correlation analysis showed that the increase in insulin content was positively correlated with increased body weight in mice, indicating that the increase in insulin secretion might be the consequence of the increased body weight in mice.

IPGTT and insulin signaling results further confirmed abnormal glucose tolerance and insulin resistance in HIF1αKOMBH mice. The higher blood glucose levels in the knockout mice after glucose injection suggested that the mediobasal hypothalamic neuronal HIF1α knockout mice caused abnormal glucose tolerance in mice. The HIF1αKOMBH mice had a significantly delayed decrease in blood glucose after glucose injection, indicating that the absence of HIF1α in the neurons of medial basal hypothalamus might lead to impaired glucose clearance in the blood of mice. The glucose intolerance in HIF1αKOMBH mice may be due to higher insulin secretion baseline in these mice, which leads to insulin deficiency when facing glucose challenge. Akt, also known as PKB or Rac, plays a critical role in the control of survival and apoptosis (23–25). Insulin promotes a variety of important biological responses (26) and can stimulate the disposal of blood glucose primarily in target tissues, such as skeletal muscle and adipocytes, where the sugar is either oxidized or stored as glycogen or fatty acids. In both tissues, insulin promotes a rapid activation of specific PKB isoforms. Our results showed that the insulin-induced activation of AKT signaling pathway in the liver, epididymal fat, and skeletal muscle of HIF1αKOMBH mice was significantly lower than that of the control mice, confirming the reduced insulin sensitivity in these mice.

To investigate the lipid metabolism of HIF1α knockout mice in the mediobasal hypothalamus, we measured the serum TG, TC, FFA, HDL, and LDL levels in HIF1α*^flox/flox^* mice after virus injection. Although there are no significant changes in the serum TG content, the serum TC and FFA content in knockout mice significantly increased. The abnormal blood lipid content still appeared in the mice under chow diet, suggesting that HIF1α in the neurons in the mediobasal hypothalamus was also involved in blood lipid metabolism to some extent. The increased HDL and LDL level may be the consequence of increased lipid level.

To confirm that the HIF1α was indeed knocked out in HIF1αKOMBH mice, the RT-PCR of HIF1α was performed. When we checked the HIF1α in the whole hypothalamus, we found that the HIF1α did not decrease. Since the virus injections are mainly in the mediobasal hypothalamus, we took the ventral part of the hypothalamus to perform the RT-PCR again. As a result, the expression of HIF1α mRNA in HIF1αKOMBH mice decreased by 74%, indicating that AAV-hSyn-cre-GFP had a knockdown effect on HIF1α. Considering the knockout is neuronal specific, 74% decrease in HIF1α expression is reasonable.

The ARC in the hypothalamus has two distinct functional types of neurons that are important for appetite regulation. These neurons are related peptide agouti-related protein (AGRP)/NPY neurons, which express food-derived (appetite-stimulating) NPY and AGRP, and POMC neurons, which express POMC and inhibit appetite (27). NPY, as a polypeptide widely distributed in the central and peripheral nervous systems, plays an important role in body weight regulation. The main role of NPY is to increase food intake and reduce the thermogenic effect of satiated animals. The RT-PCR in the whole hypothalamus suggested nonchanged expression of POMC and NPY. Interestingly, however, in ventral hypothalamus, the mRNA expression of POMC and NPY were significantly reduced in HIF1αKOMBH group. The decreased POMC expression may explain the increased food intake in knockout mice, while the decrease in NPY mRNA expression might be the result of negative feedback regulation due to overeating in mice.

Previous study found that Glut4 is also expressed in the hypothalamus (28) and plays an important role in sensing glucose and regulating systemic glucose homeostasis. The knockdown of Glut4 in the mouse brain leads to impaired glucose tolerance, reduced insulin sensitivity, and impaired glucose-lowering regulation in mice (29). Moreover, the deletion of HIF1α in skeletal muscle cells leads to impaired Glut4 function (30). By contrast, the insulin receptor substrate 1-associated phosphatidylinositol 3-phosphate kinase can activate Glut4 translocation to the plasma membrane, thereby importing glucose into the cell. Therefore, Glut4 is a key factor in skeletal muscle insulin sensitivity. The results of the present study showed a decreased Glut4 mRNA expression in HIF1αKOMBH mice in the whole hypothalamus, suggesting that Glut4 may be involved in the weight gain and abnormal glucose metabolism of these mice. However, the expression of Glut4 did not change in ventral hypothalamus, suggesting that the effect of HIF1α may act on the dorsal part of hypothalamus.

In summary, our study showed that neuronal HIF1α knockout at the mediobasal hypothalamus could lead to weight gain in mice accompanied with impaired glucose metabolism and lipid metabolism. POMC and Glut4 may be related to this effect of HIF1α. Whether the abnormal glucose metabolism and lipid metabolism was directly and causally related to the deletion of HIF1α at the mediobasal hypothalamus in mice remained unclear. In the future study, we will further investigate the effects of hypothalamic neuronal HIF1α on hypothalamic glucose sensing, insulin signaling pathway, and lipid regulatory pathway and mechanisms.

## Data Availability Statement

The raw data supporting the conclusions of this article will be made available by the authors, without undue reservation.

## Ethics Statement

The animal study was reviewed and approved by Animal Ethics Committee of Xinjiang Medical University.

## Author Contributions

AR, WS, and QY performed experiments and analyzed data. AR and QY prepared the figures and drafted the manuscript. QY conceived and designed the research and approved the final version of manuscript. All authors contributed to the article and approved the submitted version.

## Conflict of Interest

The authors declare that the research was conducted in the absence of any commercial or financial relationships that could be construed as a potential conflict of interest.

## Publisher’s Note

All claims expressed in this article are solely those of the authors and do not necessarily represent those of their affiliated organizations, or those of the publisher, the editors and the reviewers. Any product that may be evaluated in this article, or claim that may be made by its manufacturer, is not guaranteed or endorsed by the publisher.
